# Genome-wide association study of drought tolerance traits in sugar beet germplasms at the seedling stage

**DOI:** 10.3389/fgene.2023.1198600

**Published:** 2023-07-21

**Authors:** Wangsheng Li, Ming Lin, Jiajia Li, Dali Liu, Wenbo Tan, Xilong Yin, Yan Zhai, Yuanhang Zhou, Wang Xing

**Affiliations:** ^1^ National Beet Medium-Term Gene Bank, Heilongjiang University, Harbin, China; ^2^ Key Laboratory of Sugar Beet Genetics and Breeding, College of Advanced Agriculture and Ecological Environment, Heilongjiang University, Harbin, China; ^3^ Xinjiang Academy of Agricultural Sciences, Urumqi, China

**Keywords:** sugar beet germplasms, PEG-6000 drought stress, SNP, GWAS, linkage disequilibrium

## Abstract

**Introduction:**

Sugar beets are an important crop for global sugar production. Intense drought and the increasing lack of water resources pose a great threat to sugar beet cultivation. It is a priority to investigate favourable germplasms and functional genes to improve the breeding of drought tolerant plants.

**Methods:**

Thus, in this study, 328 sugar beet germplasms were used in a genome-wide association study (GWAS) to identify single nucleotide polymorphism (SNP) markers and candidate genes associated with drought tolerance.

**Results:**

The results showed that under drought stress (9% PEG-6000), there were 11 significantly associated loci on chromosomes 2, 3, 5, 7, and 9 from the 108946 SNPs filtered using a mixed linear model (MLM). Genome-wide association analysis combined with qRT-PCR identified 13 genes that were significantly differentially expressed in drought-tolerant extreme materials.

**Discussion:**

These candidate genes mainly exhibited functions such as regulating sugar metabolism, maintaining internal environmental stability and participating in photosystem repair. This study provides valuable information for exploring the molecular mechanisms of drought tolerance and improvement in sugar beet.

## 1 Introduction

Sugar beet is an important sugar crop. It has strong drought tolerance in the middle and late growth period, but weak drought tolerance at seedling stage. Most regions in the world are facing the problem of drought, and the sugar beet plant area of Heilongjiang Province in China is no exception. In spring, there are many southerly winds, large evaporation and small precipitation. The development of drought-tolerant varieties becomes more and more important (Bloch et al., 2006), and the selection and utilization of excellent drought tolerant germplasm resources and drought tolerant genes play an important role in the utilization efficiency and the improvement of agricultural production in water-shortage areas.

Mapping of quantitative trait loci (QTLs) is an effective tool often used to reveal the genetic basis of complex quantitative traits in crops (Huang et al., 2006). However, using traditional molecular markers such as restriction fragment length polymorphism (RFLP) and simple sequence repeats (SSR), only a few QTLs associated with drought stress can be identified (Jha et al., 2021; Dwiningsih et al., 2022). With the development of functional genomics and transcriptomics in recent years, a large number of genes have been found to be involved in plant drought tolerance, including protein kinases, transcription factors and some structural genes (Wang et al., 2021b). However, only a few genes have been functional validated. It remains a major challenge to accurately and effectively screen more drought stress-related genes and use them to breed drought-tolerant varieties.

Drought tolerance in plants is a complex quantitative trait that is controlled by multiple genes and involves multiple physiological and biochemical metabolic pathways. Although QTL mapping is a powerful method for detecting genomic regions associated with complex traits, the genetic effects of QTLs may not exist or may simply not be tested in different genetic backgrounds and environments (Shariatipour et al., 2021). Compared with traditional QTL, genome-wide association study (GWAS) can utilize genome-wide single nucleotide polymorphisms (SNPs) as molecular markers to analyze the genetic basis of complex traits, assisted by high-throughput genotyping platforms (Zheng et al., 2021). With the continuous development of high-throughput sequencing and high-resolution metabolic assays, as well as the development of multiple bioinformatics techniques and statistical methods to provide a basis for precise localization of complex trait gene variants, genome-wide association analysis is playing an increasingly important role in mining genetic loci for research (Zheng et al., 2021). Significant markers associated with aboveground biomass, fruit size, lodging score and leaf elongation in beans were identified through a whole genome-wide association analysis of 96 common bean germplasms. Specifically, 7 significant markers were found under irrigated conditions, and 5 significant markers were found under water deficit conditions (Hoyos-Villegas et al., 2017). In 2019, Mathew et al. used genome-wide association analysis to identify 75 significant marker-trait associations for population structure and marker traits of wheat biomass traits under drought and non-stress conditions. A total of 37 presumptive candidate genes were screened through gene annotation on IWGSC RefSeq 1.1 (Mathew et al., 2019). In recent years, the genome-wide association method has been applied in many crop drought tolerance studies and achieved good results. However, the current research on drought tolerance of sugar beet mainly focus on physiological and biochemical responses. Although traditional breeding methods such as analysis of combinin ability of lines have been used for selection of superior sugar beet genotypes for sugar related traits (Hassani et al., 2020), GWAS provide more reliable information about markers linked with sugar trait for use in marker-assisted selection We believe that using genome-wide association analysis in drought tolerance studies of beet germplasm resources can quickly and accurately excavate drought tolerance related genes. Therefore, in this study, 328 beet germplasm resources were simulated by PEG-6000 drought stress. It is hoped to explore significant loci and potential candidate genes related to drought tolerance of beet germplasm resources by genome-wide association analysis, and to provide reference and basis for further research on the isolation of related genes and molecular marker-assisted selection of beet drought tolerance.

## 2 Materials and methods

### 2.1 Test material

**FIGURE 1:**
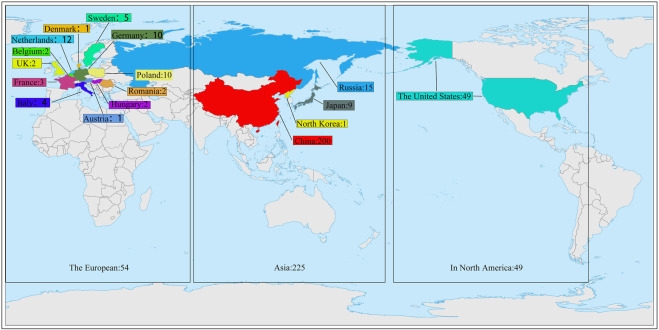
328 sugar beet germplasm resources distribution map.

The material for this study was provided by the National Beet Medium-Term Gene Bank at Heilongjiang University. The 328 sugar beet germplasm resources used in the trial were from 17 countries (Supplementary Table S1). 200 from China, 9 from Japan, 1 from North Korea, 15 from Russia, 49 from the United States, 1 from Austria, 2 from Belgium, 10 from Poland, 1 from Denmark, 10 from Germany, 3 from France, 12 from the Netherlands, 2 from Romania, 5 from Sweden, 2 from Hungary, 4 from Italy, and 2 from the United Kingdom (Figure 1). This experiment was conducted in a sophisticated climate chamber with a constant environment of 25°C and 60% humidity during the day and 18°C and 60% humidity during the night. There were 4 rows for each genotype, and 6 plants in each row were cultured in Hoagland’s solution. After growing one pair of true leaves, two of the four rows of germplasms were selected and treated with PEG-6000 (Livak and Schmittgen) at a concentration of 9% to simulate drought stress (Tan et al., 2023). The remaining two rows of germplasms continued to be cultured in Hoagland’s solution as controls (CG). The solution was changed once every 3 days. The materials were sampled after the growth of three pairs of true leaves (Cai et al., 2015).

### 2.2 Phenotype identification and physiological trait assay

The following traits were measured in this test: embryonic axis diameter (stem diameter measured using Vernier callipers) (Yang et al., 2022b), maximum root length (a straightedge was used to measure the maximum length of the root), plant height (measured from the ground to the top of the plant using a straightedge), root fresh weight (the roots were removed, dried on paper towels, and weighed on an electronic balance), leaf fresh weight (all leaves were cut off and weighed on an electronic balance), root dry weight (roots were dried in a ventilated oven at 105°C for 15 min, then transferred to 75°C for drying to a standard weight) (Polash et al., 2018), and leaf dry weight (leaves were dried in a ventilated oven at 105°C for 15 min, then transferred to 75°C for drying to a standard weight) (Yang et al., 2022a). Related tools were Vernier callipers (0.01 mm, Brand: Sheffield, Model: S071012, Origin: Mainland China), electronic balance (0.00001 g, Brand: Mettler-Toledo GmbH, Model: MS105DU/A, Origin: Switzerland), and steel ruler (0.1 cm, Brand: Sheffield, Model: S079020, Origin: China Mainland). The relative leaf water was calculated as follows:

Relative leaf water = (Leaf fresh weight-Leaf dry weight)/(Leaf saturated fresh weight-Leaf dry weight).

Superoxide dismutase activity was evaluated by the nitrogen blue tetrazolium method (Spitz and Oberley, 1989). Soluble sugar levels were evaluated by the Anthrone-colorimetric method (Maness, 2010). Proline levels were evaluated by the acidic ninhydrin method (Carillo and Gibon, 2011). Soluble protein levels were evaluated using a BCA Protein Assay Kit (Brand: biosharp, Model: BL521A-3, Origin: Beijing, China).

The drought tolerance coefficient (DTC) was calculated for each trait:

$$DTC_{X}=DT/NT$$

where DTC_X is the drought tolerance coefficient of each trait; DT is the mean value of the traits under drought treatment; and NT is the mean value of the traits under normal treatment.

### 2.3 Genotype data

Sequencing: SNPs were identified by NOVOGENE (Beijing, China) and sequenced using the Illumina HiSeq sequencing platform with double-end (Paired-End) 150 sequencing. BWA (Li and Durbin, 2009) (0.7.17): Interference information was filtered out of the raw data to obtain high-quality clean data, and effective high-quality sequencing data were aligned to the reference genome by BWA software (parameter: mem-t 4-k 32-M) (reference genome download address: ftp://ftp.ncbi.nlm.nih.gov/genomes/all/GCF/000/511/025/GCF_000511025.2_RefBeet-1.2.2/GCF_000511025.2_RefBeet-1.2.2_genomic.fna.gz). Samtools (Soskic et al., 2019) (1.9): Samtools was used to transform the format of the sam file and build an index to generate the bai file. GATK (Sun et al., 2019) (4.2.6.1): GATK was used to detect mutations and generate group modification.vcf file. VCFtools (Ratnapriya et al., 2019) (0.1.17): A total of 108946 high-quality loci were screened for subsequent analysis using sequencing depth (dp ≥ 4), minor allele frequency (maf ≥ 0.05) and miss rate (miss ≤ 80%).

### 2.4 Population structure and LD analysis

VCFtools (0.1.17) was first used to convert the.vcf files ped files and then plink (1.9) software was used to convert ped format to bed format (Cadzow et al., 2014). Population structure analysis of bed files was performed using admixture (1.3.0) software, the population size K value was preset to 1–10 for classification, and the minimum value of cross entropy was used to determine the optimal number of classifications (Byrne et al., 2020). Principal component analysis of 108946 high-quality SNP loci was performed using Tassel (5.2.82) software (Breria et al., 2020), and the PCA results were visualized using R (4.1.0) software. The LD of 328 sugar beet germplasms was analysed by PopLDdecay (3.4.2). The LD intensity (r2) between two SNPs within a certain distance was calculated (Xu et al., 2018).

### 2.5 Genome-wide association study

The kinship analysis was performed using Tassel (5.2.82) software to obtain the kinship matrix (Zeng et al., 2017) and the results were visualized using the pheatmap package in R (4.1.0). The mixed linear model (MLM) (Kang et al., 2015) of Tassel software was used to analyse the PCA and kinship analysis results as covariates. This combination (Q + K) is often considered the most powerful, and this model can classify crops into different subgroups based on growth habit or geographical origin. Mixed models corrected with population structure and kinship can effectively reduce false positive results (Wang et al., 2007). Manhattan and Q-Q plots were visualized using the CMplot package of R (4.0.1) (Kumar et al., 2021). The mixed linear model (MLM) equation was as follows:

Y=Xα+Qβ+Kμ+e

In the equation, Y is the vector of measured phenotypes, X is the fixed effect of SNPs, P is the fixed effect of population structure, K is the random effect of kinship, and e is the random error (Dramadri et al., 2021).

We used the powerful annotation function of the internationally recognized UniProt database (Yang and Wang, 2015) to annotate the SNPs. After screening fake SNPs according to genome annotation, we obtained high-quality and significant SNPs with a threshold of −log10p> 6.

The SNPs were screened to obtain high-quality and significantly correlated SNPs with a threshold of −log10p> 6. The size of the screened SNP interval was estimated based on LD block analysis, and genes in the region near the locus were obtained and functionally annotated. Based on the functional annotations, we could roughly determine which genes might be associated with the traits of interest and use in subsequent analysis (Bai et al., 2022).

### 2.6 Real-time fluorescence quantitative PCR

Four drought-tolerant (Mono HY53, Neitang578, Gansunongjiazhong, BGRC16137) and four drought-sensitive germplasms [7501A/BCE, 92017/1-8/1, Tianyansanhao (X), 92017/1-4/1] were used as extreme materials to extract total RNA according to the Trizol method. cDNA was synthesized according to the TranScript One-Step gDNA Removal and cDNA Synthesis SuperMix protocol (AT311, Beijing All Style Gold Biotechnology Co., Ltd.). qPCR was performed using the SuperReal PreMix Plus kit (Beijing Tiangen Biochemical Technology Co., Ltd., version FP210831) on a real-time fluorescent quantitative PCR instrument (Thermo Fisher Scientific Instruments, Shanghai. QuantStudio™ 1 Plus) (Liu et al., 2023). Expression level of 14 genes was analyzed with the 2^−ΔΔCT^ method (Livak and Schmittgen, 2001). *BvGAPDH* (NC_ 024800) was used as an internal control to standardize the expression levels of the different samples. All assays were performed in two independent experiments and replicated three times. Primers were summarized in Supplementary Table S5.

## 3 Results

### 3.1 Phenotypic and physiological analysis

**FIGURE 2:**
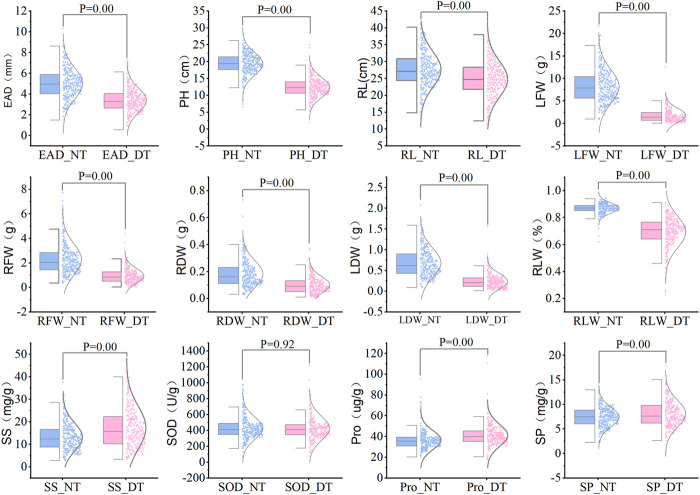
Comparison of various traits between drought treatment and normal water supply for 328 sugar beet germplasms. Note: NT, normal treatment; DT, drought treatment, 9% PEG-6000 for 7 days.

The statistical results showed that the measured data were all close to a normal distribution, indicating that the 12 traits were all typical quantitative traits. Eight traits, including embryonic axis diameter (EAD), plant height (PH), root length (RL), leaf fresh weight (LFW), root fresh weight (RFW), root dry weight (RDW), leaf dry weight (Lander et al., 2001) and leaf relative water content (RLW), were significantly lower in the drought-treated plants than in the control plants. Three indicators, including soluble sugar levels (SS), soluble protein levels (SP) and free proline levels (Pro), were significantly higher in the drought-treated plants than in the control plants (Figure 2). Drought tolerance coefficients for each trait showed that these traits all exhibited suitable variation, indicating that they were controlled by small effect polygenes with coefficients of variation (CV) ranging from 16.29% to 79.58% (Table 1). The CV is an absolute value reflecting the degree of dispersion of each trait in the population. To better estimate the correlations between markers and traits, it is desirable to use highly dispersed trait data for statistical analysis. The traits in this test exhibited a high degree of dispersion and were suitable for genome-wide association analysis.

**TABLE 1:** Analysis of variation in drought tolerance coefficient of 328 sugar beet germplasm for each trait.

Traits	Mean	STDEV	Max	Min	Skewness	Kurtosis	CV(%)
EAD	0.708	0.243	1.676	0.171	0.718	0.848	34.38
PH	0.651	0.154	1.197	0.259	0.506	0.586	23.58
RL	0.914	0.215	1.659	0.475	0.549	0.238	23.55
LFW	0.236	0.188	1.042	0.005	1.411	2.112	79.58
RFW	0.482	0.322	1.879	0.015	1.269	1.87	66.73
RDW	0.634	0.406	2.333	0.022	1.261	1.923	64.16
LDW	0.394	0.278	1.684	0.019	1.341	2.74	70.62
RLW	0.799	0.130	1.226	0.267	−1.062	2.581	16.29
SS	1.611	1.225	6.943	0.152	1.725	3.249	76.03
SOD	1.023	0.263	1.994	0.204	0.532	1.409	25.72
Pro	1.156	0.319	2.185	0.362	0.539	0.446	27.57
SP	1.130	0.415	2.715	0.006	0.831	0.928	36.70

Note: EAD, embryonic axis diameter; PH, plant height; RL, maximum root length; LFW, leaf fresh weight; RFW, root fresh weight; RDW, root dry weight; LDW, leaf dry weight; RLW, relative leaf water; SS, soluble sugar levels; SOD, superoxide dismutase activity; Pro, Proline levels; SP, soluble protein levels.

### 3.2 Population structure and genetic diversity

**FIGURE 3:**
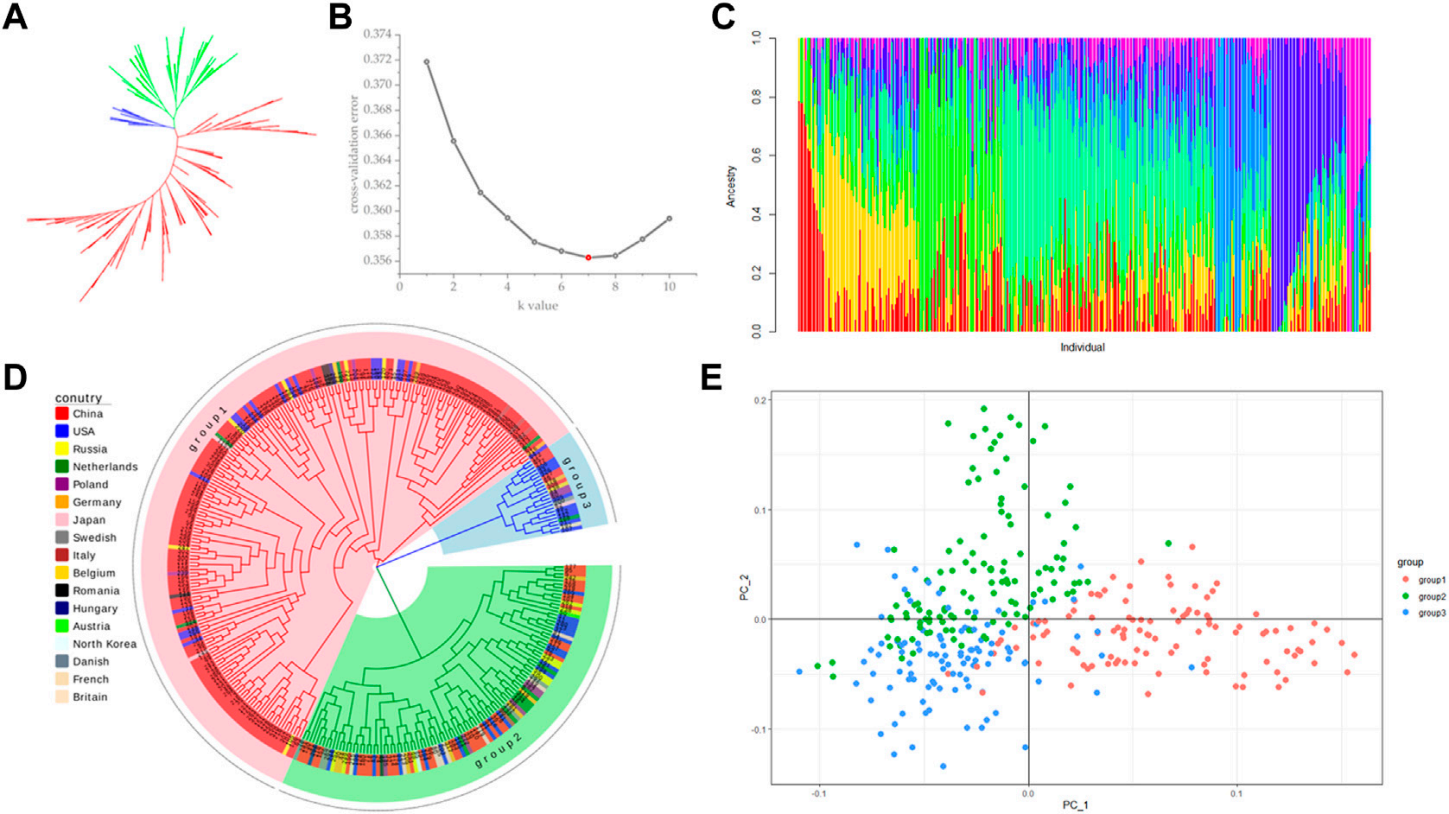
Population structure analysis. **(A)** NJ method unrooted evolutionary tree; **(B)** cross-validation (CV) error value; **(C)** estimation of population structure; **(D)** NJ method rooted evolutionary tree; **(E)** principal component analysis (PCA).

Population stratification refers to the existence of subgroups within a population, where the diversity among individuals within the subgroups is greater than the average diversity among individuals within the entire population (Wang et al., 2021a). Different allele frequencies at certain loci between different subpopulations can lead to false positive results when using two subpopulations for association analysis (Zheng et al., 2021). Therefore, before performing association analysis, we used a subset of 108946 SNPs without close linkage for population structure prediction. Using Tassel (5.2.82) software to construct phylogenetic trees by the neighbor-joining (NJ) method, 328 sugar beet germplasms were divided into three subgroups, but the differences between the subgroups were small (Figures 3A, D). The results from a principal component analysis (PCA) of the above three subgroups were consistent with the phylogenetic analysis, and no significant grouping was found (Figure 3E). The results indicated that the 328 germplasms without significant grouping were suitable for a GWAS. Using admixture software, the number of subgroups (K) was set to 1–10, and each K value was repeated three times to select the optimal K value according to the maximum likelihood method. The cross-validation error (CV error) value was the smallest when K = 7 (Figure 3B), so the Q-matrix with K = 7 was used as a covariate to improve the accuracy of the GWAS (Figure 3C).

**FIGURE 4:**
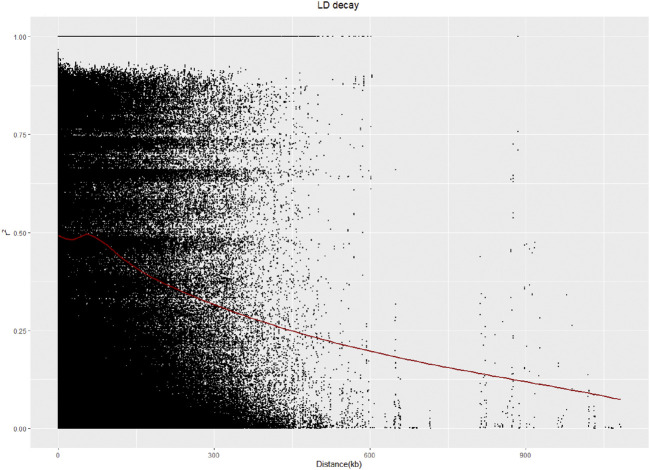
LD analysis of 328 sugar beet germplasms.

A linkage disequilibrium (LD) analysis was carried out, and the LD distance decreased as the physical location of the SNPs on chromosomes gradually increased. The LD distance of 50 kb was equal to half of the maximum value (Figure 4).

**FIGURE 5:**
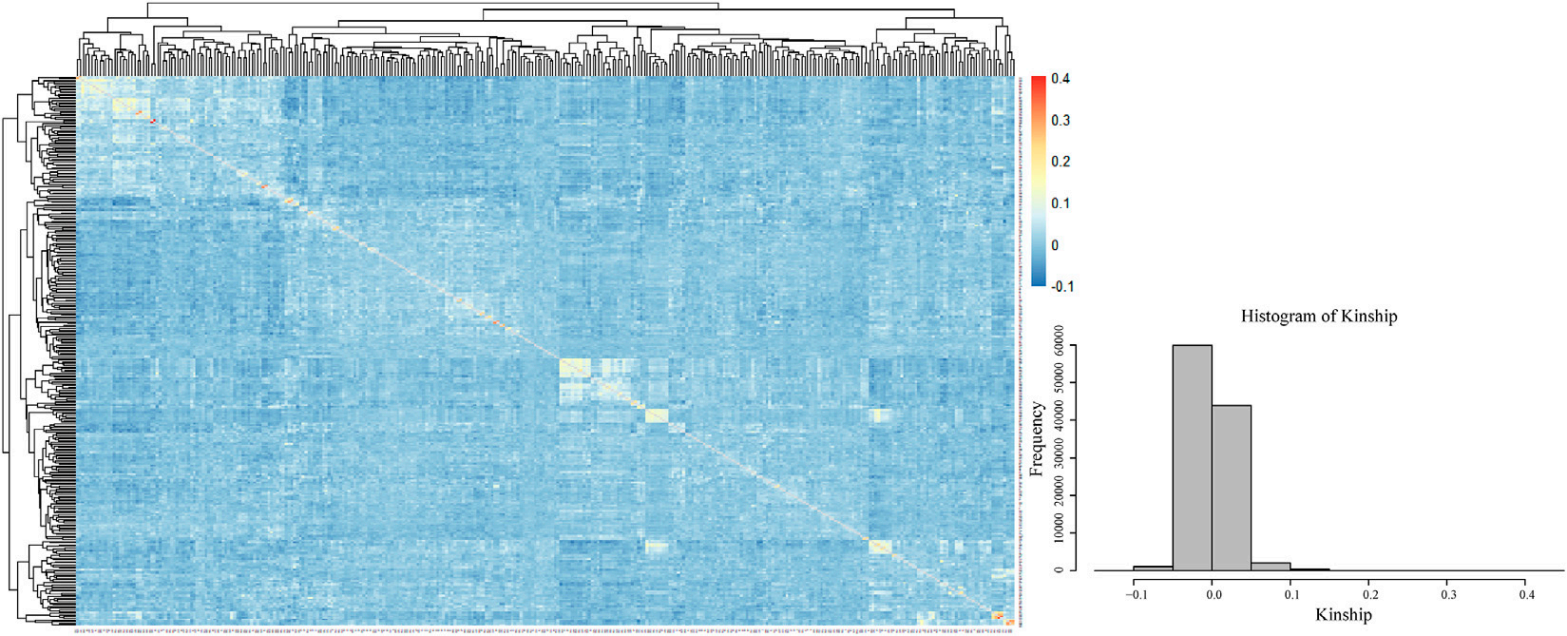
Kinship heatmap and cluster analysis.

The visualization of the kinship matrix showed that the variation between most of the 328 genotypes was low (light blue), and the genetic similarity between individual genotypes was high. The low genetic variation in this study would reduce the occurrence of false positive results (Figure 5).

### 3.3 GWAS of drought tolerance coefficients for each trait

**FIGURE 6:**
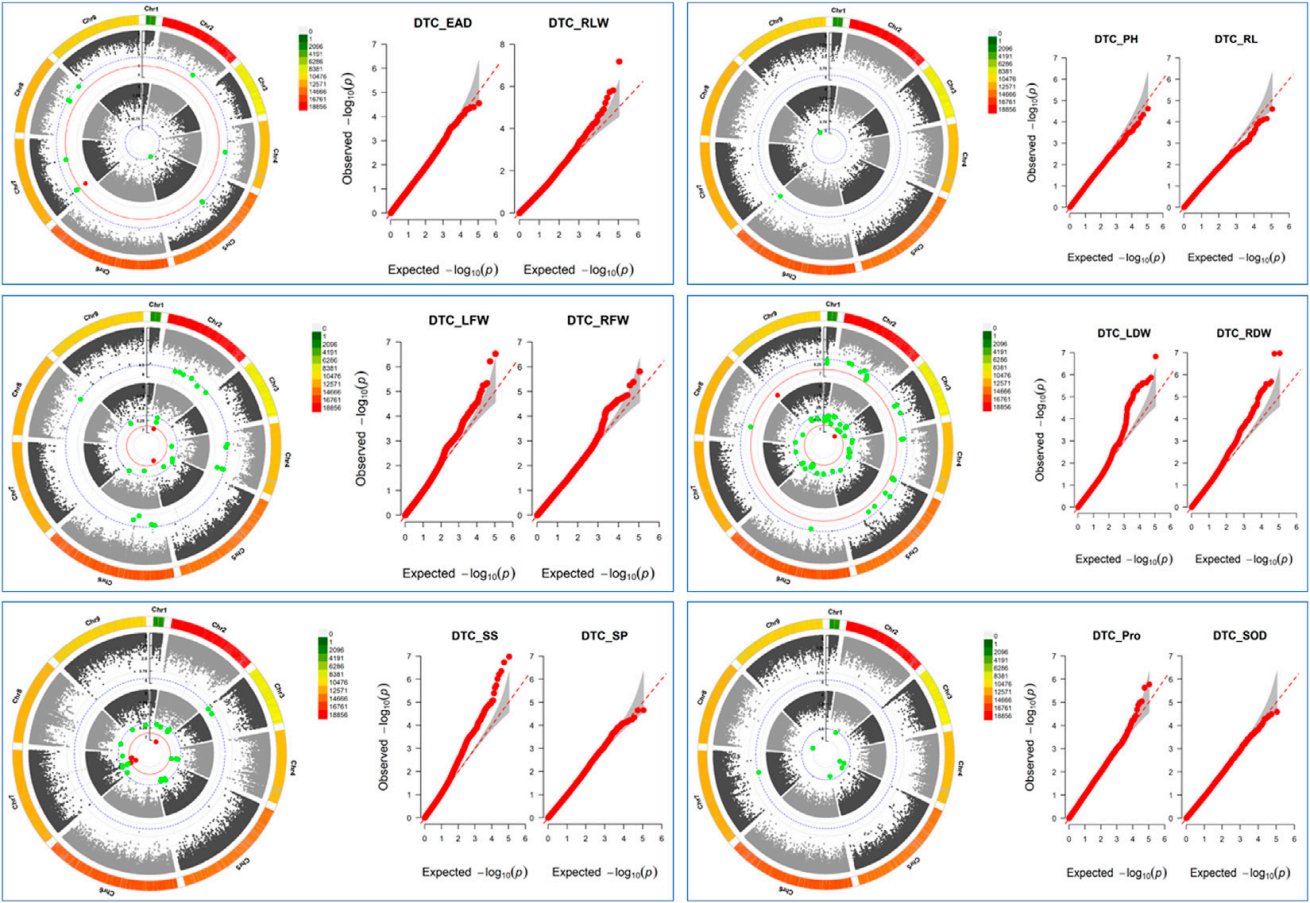
Manhattan plot: inner circles indicate single nucleotide polymorphisms (SNPs) associated with the Q-Q plot on the left, outer circles indicate single nucleotide polymorphisms (SNPs) associated with the Q-Q plot on the right, grey dashed lines indicate SNP loci with −log10p = 4.5, red dashed lines indicate SNP loci with −log10p = 6, and the outermost side indicates SNP density on the chromosome. Q-Q plots: horizontal coordinates indicate expected values, vertical coordinates indicate observed values, and grey areas are 95% confidence intervals.

The GWAS results showed (Figure 6; Supplementary Table S2) that a total of 183 SNPs (−log10p > 4.5) were identified in the 12 drought tolerance-related traits, including 11 SNPs with peaks >6.0, which the study mainly focused on. It was found that 1 SNP on chromosome 7 was associated with DTC_RLW; 2 SNPs associated with DTC_LFW were located on chromosomes 2 and 5; 1 SNP on chromosome 3 was associated with DTC_LDW; 2 SNPs on chromosome 9 were associated with DTC_RDW; and there were 5 SNPs associated with DTC_SS on chromosomes 2 and 7. It is worth noting that the two significant loci associated with DTC_RDW were close to each other with a distance of only 216 bp.

### 3.4 Candidate genes associated with sugar beet drought tolerance

In the five traits (−log10p> 6) of DTC_RLW, DTC_LFW, DTC_LDW, DTC_RDW, and DTC_SS, 50 kb upstream and downstream of the significant loci were analyzed by LD decay. A total of 24 genes in sugar beet associated with drought tolerance were identified and annotated (Supplementary Table S3).

**FIGURE 7:**
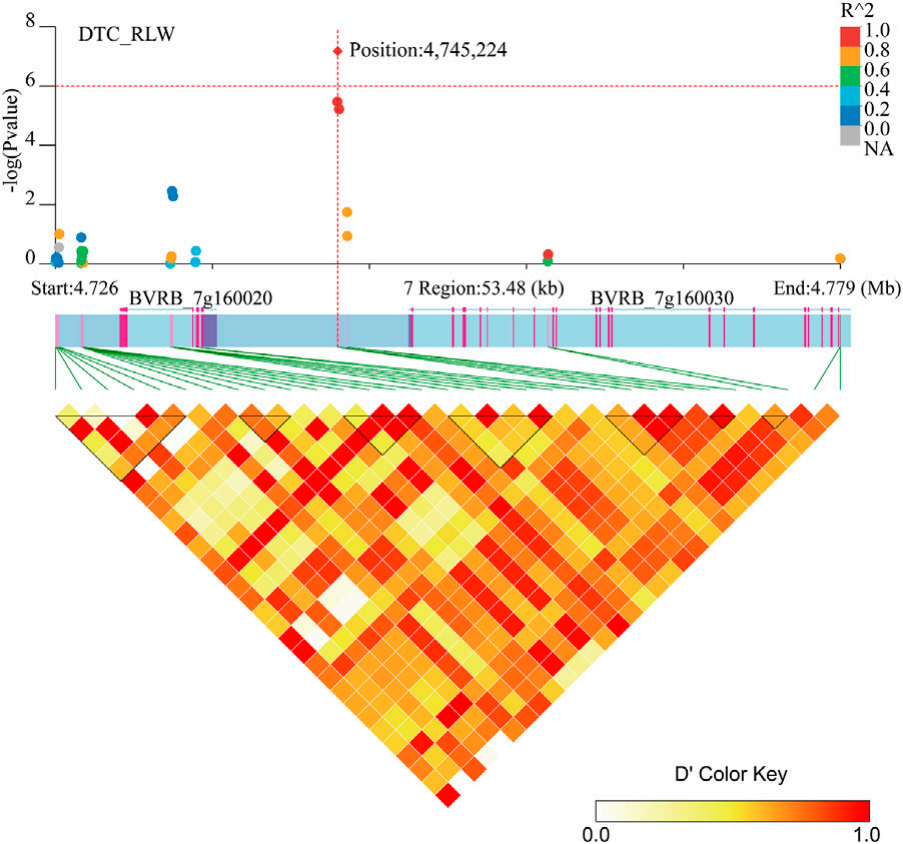
Manhattan plot of candidate gene regions of DTC_RLW and heatmap of LD (bottom). The orange vertical line indicates the position of the most significant SNPs, and the orange horizontal line indicates −log10p = 6.

Two genes (*BVRB_7g160020* and *BVRB_7g160030*) were identified on chromosome 7 at position 4,745,224 may be associated with DTC_RLW (Figure 7; Supplementary Table S3). *BVRB_7g160020* encodes a tyrosine-protein phosphatase, RLPH2, which is reported to be a new phospho-tyrosine-specific phosphatase belonging to the phosphoprotein phosphatase (PPP) family (Uhrig et al., 2016). *BVRB_7g160030*, as 1,4-alpha-glucan-branching enzyme 2-2, is mainly involved in amylose synthesis in gluconeogenesis (Han et al., 2022). It was reported that *BVRB_7g160030* catalytically regulates *α* (1–6) glycosidic bond branching synthesis. We believe that *BVRB_7g160030* is a potential target gene for the drought stress response in sugar beet.

**FIGURE 8:**
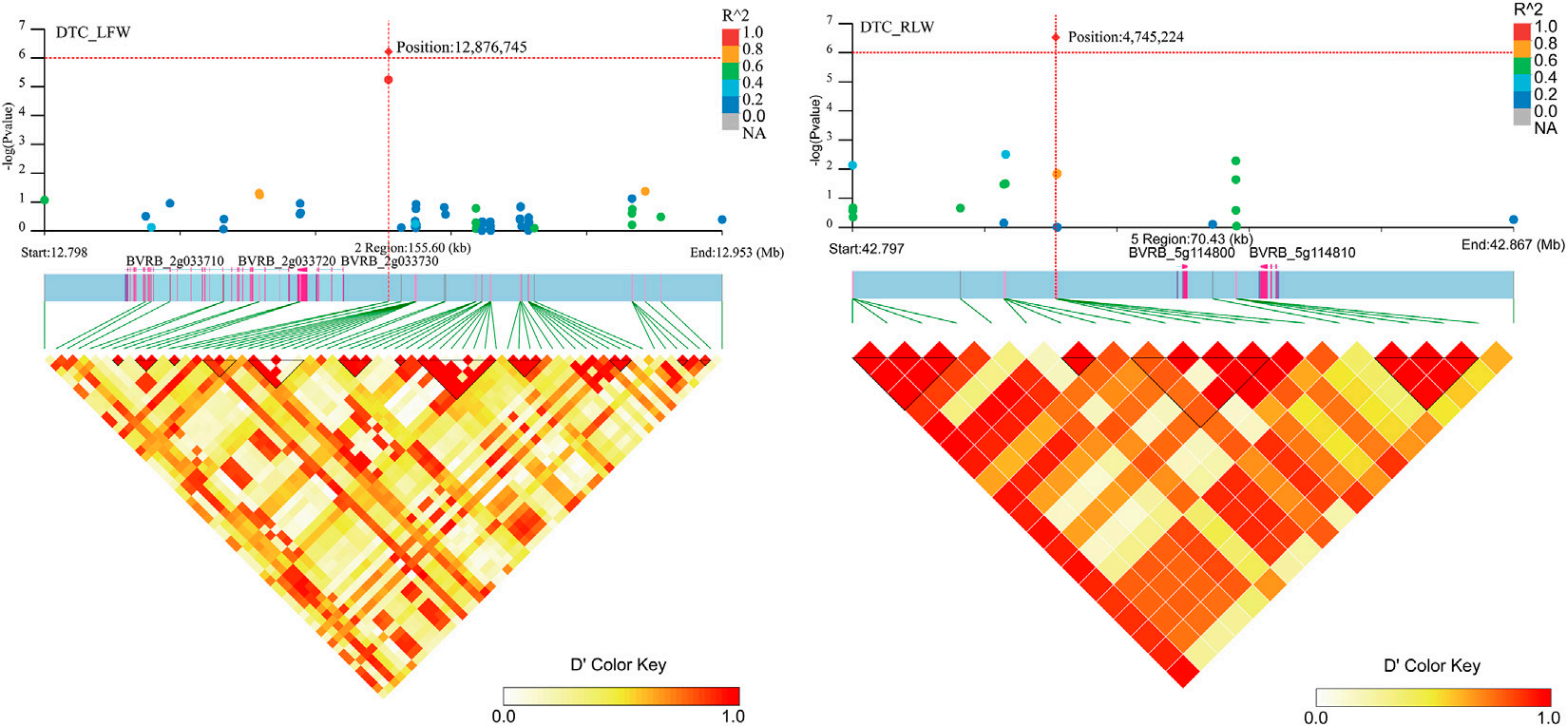
Manhattan plot of candidate gene regions associated with DTC_RLW and heatmap of linkage disequilibrium (bottom). The orange vertical line indicates the position of the most significant SNP, and the orange horizontal line indicates −log10p = 6.

Associated with DTC_LFW, three genes (*BVRB_2g033710*, *BVRB_2g033720* and *BVRB_2g033730*) were found on chromosome 2. Two genes (*BVRB_5g114800* and *BVRB_5g114810*) were found on chromosome 5. *BVRB_2g033720*, *BVRB_2g033730* and *BVRB_5g114800* were of unknown function (Figure 8; Supplementary Table S3). Through annotation, we found that *BVRB_2g033710* functions as myosin-15; *BVRB_5g114810* functions as MYB-related protein 306, the MYB structural domain is a peptide segment of approximately 51-52 amino acids containing a series of highly conserved amino acid residues and spacer sequences, and the MYB gene family is widely involved in plant metabolic regulation (Jiao et al., 2021; Johnson et al., 2022).

**FIGURE 9:**
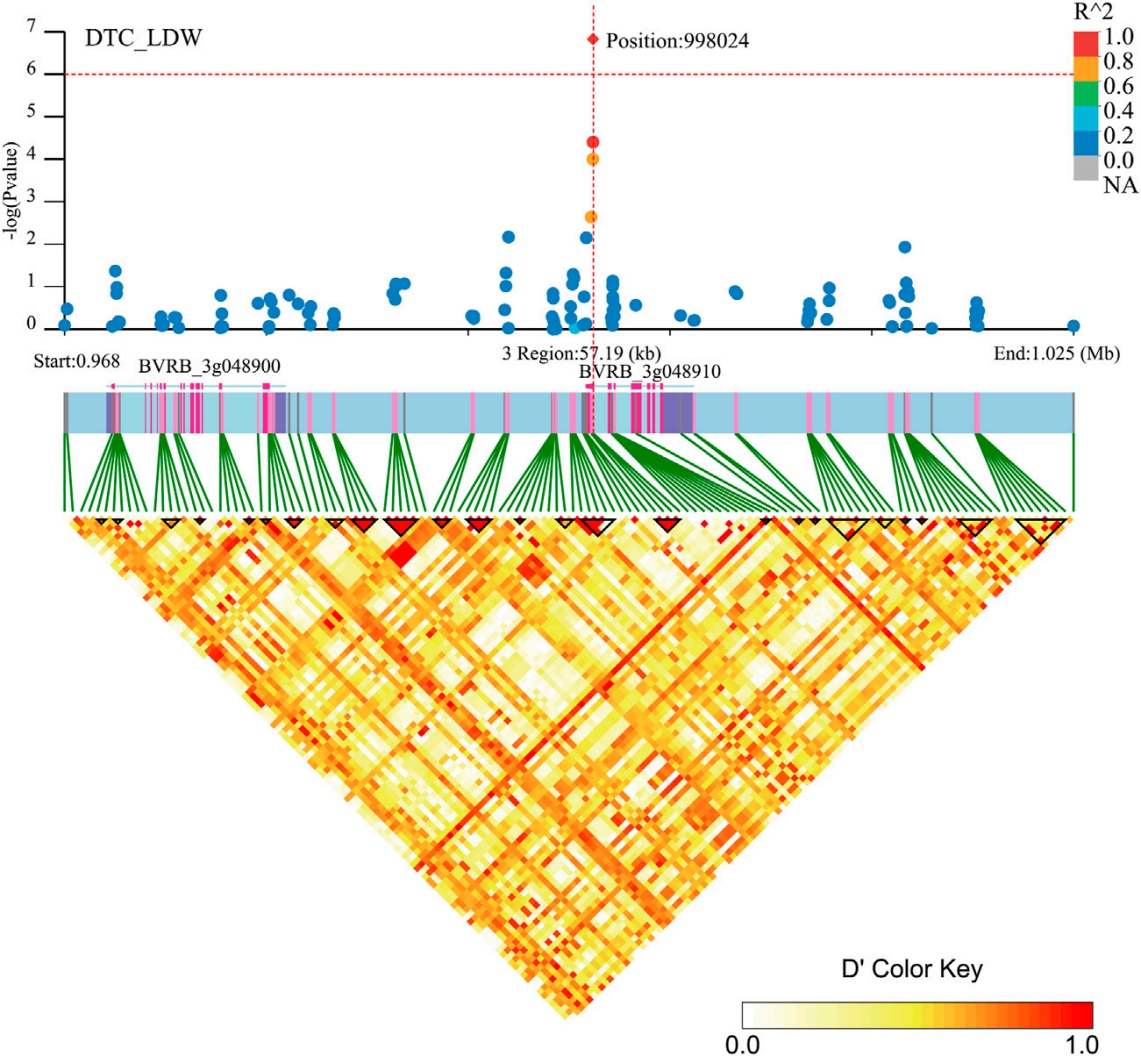
Manhattan plot of candidate gene regions associated with DTC_LDW and heatmap of linkage disequilibrium (bottom). The orange vertical line indicates the position of the most significant SNP, and the orange horizontal line indicates −log10p = 6.

Two genes (*BVRB_3g048910* and *BVRB_3g048900*) were identified on chromosome 3 position: 998,024, may be associated with DTC_LDW (Figure 9; Supplementary Table S3). By annotation, we found that *BVRB_3g048910* functions as the probable methyltransferase PMT3. Methyltransferases use S-adenosylmethionine, betaine and dimethylthetin as methyl donors to generate methionine. *BVRB_3g048900* functions as serine/threonine-protein kinase tricornered (TOR), which functions as a phosphate donor using ATP to phosphorylate serine and threonine residues on proteins to regulate abscisic acid, growth hormone, glucose and sucrose-mediated signalling (Ahn et al., 2011; Xiong et al., 2013; Deng et al., 2016).

**FIGURE 10:**
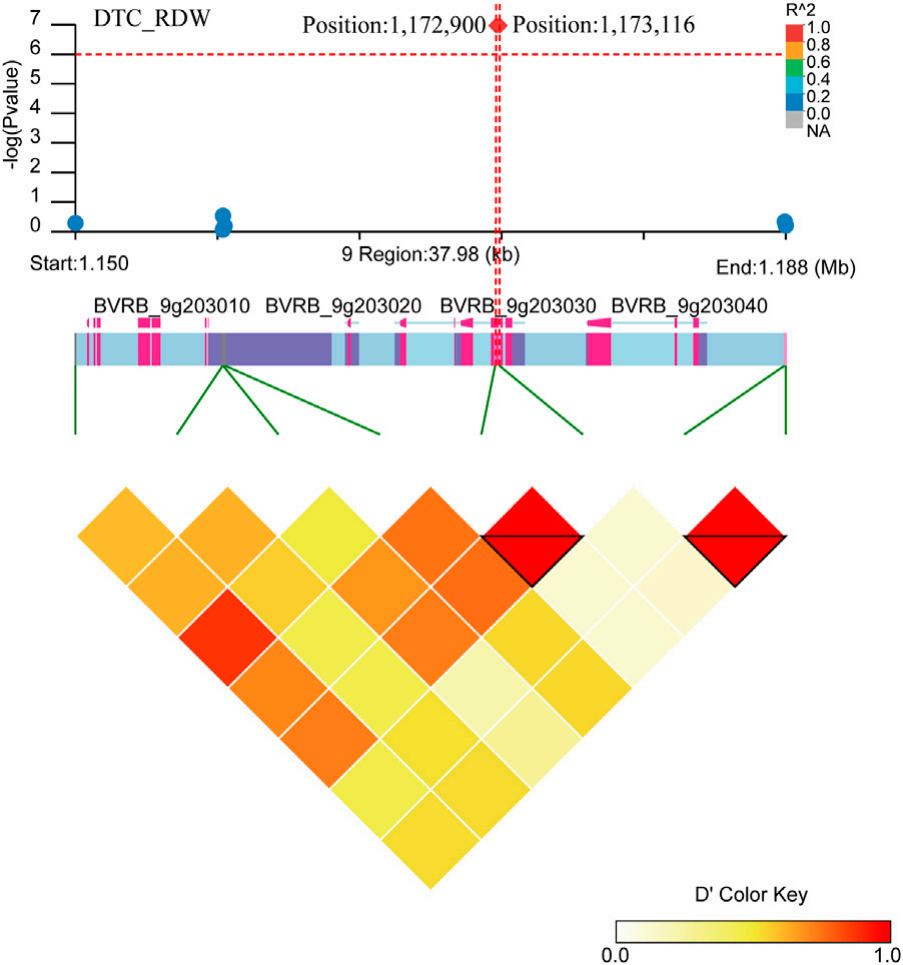
Manhattan plot of candidate gene regions associated with DTC_RDW and heatmap of linkage disequilibrium (bottom). The orange vertical line indicates the position of the most significant SNP, and the orange horizontal line indicates −log10p = 6.

Two close significant loci (position: 1,172,900, position: 1,173,116) on chromosome 9 were found to be associated with DTC_RDW (Figure 10; Supplementary Table S3). Six gene were found in the region near these two significant loci (*BVRB_9g203050*, *BVRB_9g203040*, *BVRB_9g203030*, *BVRB_9g203020*, *BVRB_9g203010* and *BVRB_9g20300*). *BVRB_9g203050* functions as photosystem II (PSII) stability/assembly factor HCF136 and is essential for PSII biogenesis. BVRB_9g203030 functions as eukaryotic initiation factor 4A-9, which promotes the hydrolysis of ATP and drives RNA deconvolution against abiotic stress; *BVRB_9g203000* functions as enhancer of AG-4 protein 2, which possesses the dual ability to induce apoptosis and autophagy (Kumar et al., 2015), and is a transcription factor that acts as a flowering repressor by enhancing the expression of genes that delay flowering and suppressing nutritional growth to inhibit reproductive development (Wang et al., 2007).

**FIGURE 11:**
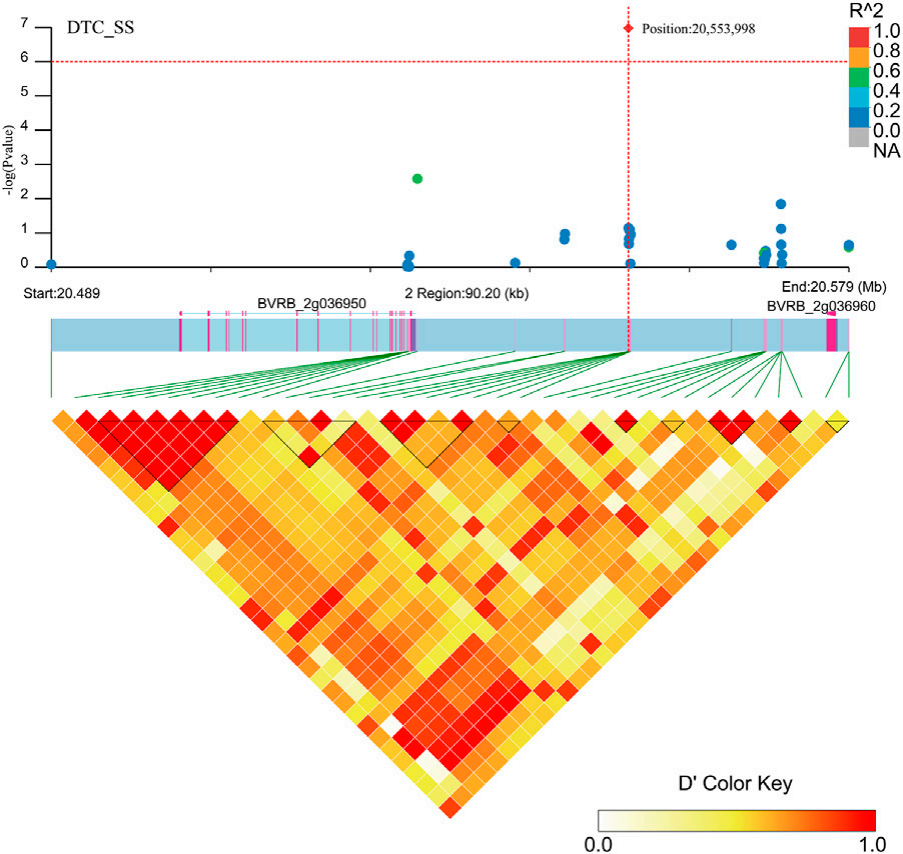
Manhattan plot of candidate gene regions associated with DTC_SS and heatmap of linkage disequilibrium (bottom). The orange vertical line indicates the position of the most significant SNP, and the orange horizontal line indicates −log10p = 6.

Two gene regions (*BVRB_2g036960* and *BVRB_2g036950*) were found to be associated with DTC_SS (Figure 11; Supplementary Table S3) in the region near positions 20,553,998 on chromosome 2. *BVRB_2g036950* is an uncharacterized protein, and *BVRB_2g036960* functions as a probable membrane-associated kinase regulator 4. Tyrosine phosphorylation controls the activation of brassinosteroid receptors through membrane-released protein kinase inhibitors.

**FIGURE 12:**
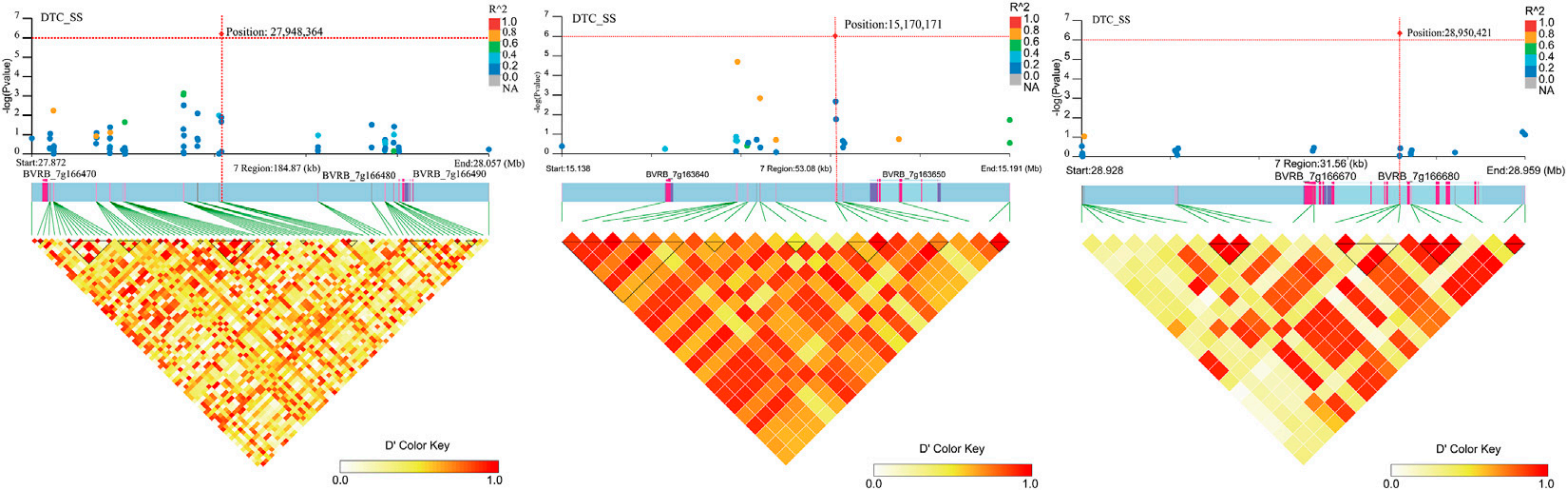
Manhattan plot of candidate gene regions associated with DTC_SS and heatmap of linkage disequilibrium (bottom). The orange vertical line indicates the position of the most significant SNP, and the orange horizontal line indicates −log10p = 6.

Four SNP loci significantly associated with DTC_SS (Figure 12; Supplementary Table S3) were found on chromosome 7. No genes were found near position 15,215,427. Two genes (*BVRB_7g163640* and *BVRB_7g163650*) were found near position 15,170,171 and they are hypothetical and uncharacterized proteins. Three genes (*BVRB_7g166470*, *BVRB_7g166480* and *BVRB_7g166490*) were found near position 27,948,364, and *BVRB_7g166470* functions as glycine-rich cell wall structural protein 1, a process that results in the assembly, arrangement of constituent parts, or disassembly of the cell wall. *BVRB_7g166480* functions as PROTEIN LOW PSII ACCUMULATION 1, which is mainly involved in the efficient assembly of photosynthetic system II. BVRB_7g166490 functions as photosystem I reaction centre subunit IV. Two gene (*BVRB_7g166670* and *BVRB_7g166680*) were found near positions 28,950 and *BVRB_7g166670* is an uncharacterized protein, and *BVRB_7g166680* functions as a ras GTPase-activating protein-binding protein.

### 3.5 qRT-PCR validation of candidate gene expression

**FIGURE 13:**
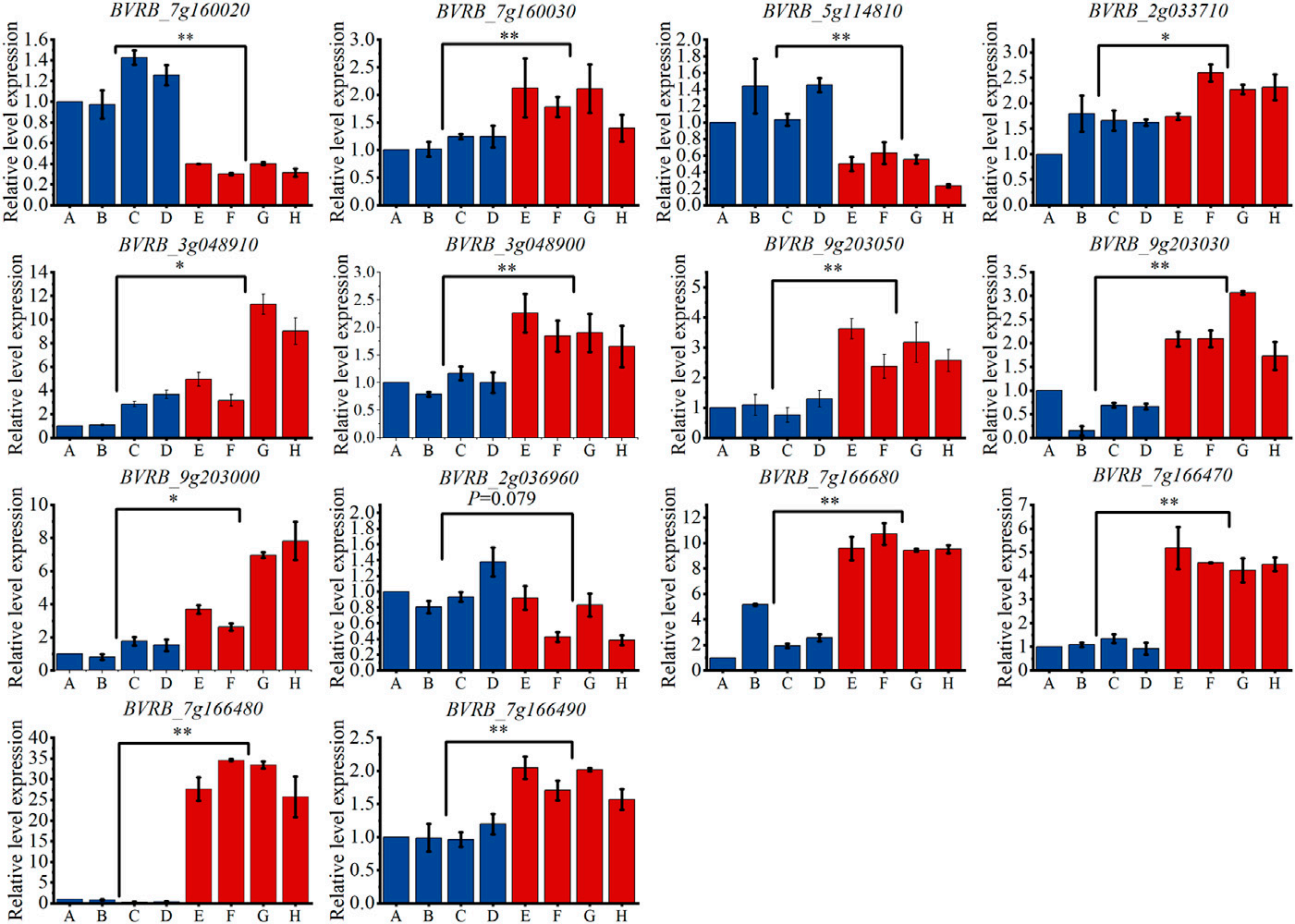
Expression levels of candidate genes in drought-tolerant extreme materials. Note: **(A–D)** are drought-sensitive materials. **(E–H)** are drought-tolerant materials. *: Indicates that the expression levels of the two groups of materials are different at the 0.05 level. **: Indicates that the expression levels of the two groups of materials are different at the 0.01 level.

By examining the overlap of the identified candidate genes with known drought stress response genes in other crop species, we identified 14 genes that may be involved in the drought tolerance process in sugar beet. In order to validate the GWAS results, qRT-PCR was performed on these genes (As shown in Figure 13). The results showed that, except for *BVRB_2g036960*, the other 13 genes were all significantly differently expressed between the drought tolerant and sensitive groups. The expression levels of *BVRB_7g160020* and *BVRB_5g114810* were 3.30-fold and 2.57-fold higher in the drought-sensitive group than them in the drought-tolerant group, respectively. *BVRB_7g160030*, *BVRB_2g033710*, *BVRB_3g048910*, *BVRB_3g048900*, *BVRB_9g203050*, *BVRB_9g203030*, *BVRB_9g203000*, *BVRB_7g166680*, *BVRB_7g166470* and *BVRB_7g166490* in drought-tolerant group were expressed 1.47-4.23-fold of them in the drought-sensitive group. Notably, the expression level of *BVRB_7g166480* in the drought-tolerant group was 46.69-fold higher than that of the drought-sensitive group.

## 4 Discussion

### 4.1 Drought tolerance-related traits in sugar beet

Drought stress is the main abiotic stress factor that causes crop yield reduction worldwide. The sugar beet plant is more drought tolerant in the middle and late growth stages but is drought sensitive in the seedling stage, which causes great taproot loss (Fugate et al., 2018). Enhancing the drought tolerance of seedlings will greatly help to improve the sugar beet crop yield. Sadeghian and Yavari conducted mannitol-simulated drought stress on 9 sugar beet varieties, assessed the dry weight of cotyledons, fresh weight of cotyledons, RFW, RDW, and RL, and concluded that the drought tolerance traits of sugar beet seedlings can be used for selective breeding of the strain with the strongest tolerance (Sadeghian and Yavari, 2004). Islam et al. (2020) screened 7 beet varieties suitable for drought conditions by carrying out drought stress experiments on 11 varieties and measuring related physiological and biochemical traits. Through stepwise regression analysis, Moosavi et al. (2017) found that root and petiole dry weight were suitable as standard traits for drought tolerance of sugar beet, and 7221 drought tolerance genotypes were screened by these traits.

Although breeding by screening drought-tolerant varieties of sugar beet can improve drought tolerance in new varieties, this method has some limitations. The transfer of agronomic traits is easily limited by intermediate reproductive isolation, which is not conducive to genetic improvement of selected crops using genetic resources of closely or distantly related species. This study aims to improve the efficiency and precision of drought-tolerant breeding of sugar beet through research methods at the molecular level.

### 4.2 Genome-wide association analysis of drought tolerance-related traits in sugar beet

With the development of genotyping technology, SNP markers are characterized by high throughput, low cost and high accuracy. The application of genome-wide association analysis is universal, and it can be used to analyze both qualitative and quantitative traits (Alqudah et al., 2020). In 175 rice samples, 13 significant SNPs were screened out and 50 genes were identified, and 10 of these genes were associated with drought and/or other abiotic stress tolerance (Pantaliao et al., 2016). It was detected 132 single nucleotide polymorphisms in sesame under drought stress, and 13 potential candidate genes were found to be related with drought tolerance (Li et al., 2018). At present, GWAS has been reported in the drought tolerance studies of many crops, but it is rarely reported in sugar beet. In this study, we explored the functional genes related to drought tolerance of sugar beet through genome-wide association analysis. 328 sugar beet germplasms were analyzed, and phylogenetic tree and PCA analysis showed that there was no obvious population stratification, indicating that the heredity of sugar beet used here was less affected by geographical factors. Considering that MLM model can better control false positive results, we used this model to excavate significant sites (Zhang et al., 2010).

### 4.3 Potential candidate genes associated with sugar beet drought tolerance

A total of 11 SNPs loci were identified by genome-wide association analysis and 25 potential candidate genes were screened out. It was found that 14 genes had been reported to be related with drought tolerance. We conducted qRT-PCR to verify gene expression levels in the two groups of drought tolerance extreme materials and found that only *BVRB_2g036960* had no difference in expression between the two groups of materials, and the expression levels of 2 genes in the drought sensitive group were significantly higher than those in the drought tolerant group. The expression levels of 11 genes in drought-tolerant group were significantly higher than those in drought-sensitive group. Therefore, these 13 differentially expressed genes might be key genes which can be used in our next research on drought tolerance in sugar beet.

*BVRB_7g160020* regulated RLPH2 is a tyrosine phosphorylase of the PPP family, and tyrosine phosphorylation is essential for signaling at the cytoplasmic structural domain (Mühlenbeck et al., 2021). We also found that the gene *BVRB_7g160030*, which is adjacent to the gene *BVRB_7g160020*, is involved in straight-chain starch synthesis in sugar metabolism. *α* (1–6) glycosidic bond branching increases the water solubility of glycogen for storage and increases the number of nonreducing ends, making it easier for biological organisms to aggregate when glycogen is needed for energy supply, and the 1,4-alpha-glucan-branching enzyme regulates glycogen metabolism and participates in the abiotic stress response (Li et al., 2019).

We found that *BVRB_7g166470* is involved in the cell wall composition. *BVRB_3g048910* can regulate methionine, which can mitigate membrane damage by peroxides. It has been shown that the probable methyltransferase PMT3 is involved in the synthesis of phosphatidylcholine (PC), which is an important precursor of lipid signalling (Baldessari and Iglesias, 2012) and a ligand for regulatory proteins (Inatsugi et al., 2009). PC appears to be a major contributor to the adaptive response of plants to environmental stress, and it plays an important role in maintaining cell membrane integrity in complex plant species. The ras GTPase-activating protein-binding protein 2 associated with *BVRB_7g166680* regulates ascorbic acid, which has strong reducing properties that effectively mitigate peroxide damage (Khazaei et al., 2020). *BVRB_9g203030* is eukaryotic initiation factor 4A-9, which promotes ATP hydrolysis to provide energy for material transport and signaling in plants, and myosin, which is a component of the cytoskeleton to provide energy for organelle movement, material transport and apical growth (Han et al., 2021). *BVRB_2g033710* is involved in the components of the cytoskeleton that provide energy for organelle movement, material transport and apex growth (Han et al., 2022). *BVRB_3g048900* on chromosome 3 was found to regulate abscisic acid, and *BVRB_5g114810* on chromosome 5 was related to MYB transcription factors. Abscisic acid can regulate MYB, which can promote lateral root growth, enhance root water absorption, and induce old leaf death to reduce water loss under drought conditions (Moosavi et al., 2017). The ENHANCER OF AG-4 protein regulated by *BVRB_9g203000* can induce apoptosis and prolong nutritional growth (Moosavi et al., 2017; Alqudah et al., 2020).

*BVRB_9g203050* on chromosome 9 regulates HCF136, which is essential for PSII biogenesis and stability (Meurer et al., 1998); the HCF136 protein is essential for the formation of early intermediates in PSII assembly, including D2 (psbD) and cytochrome b559 (Plücken et al., 2002). D2 can repair PSII and D1 expression dysfunction and maintain the stability of photosynthesis (Kanamaru and Tanaka, 2004). Protein LOW PSII ACCUMULATION (BVRB_7g166480), a required chaperone for efficient PSII assembly, is a component of the chloroplast cyst-like membrane; it binds to psbA during the biogenesis of PSII and plays an important role in repairing PSII damage to maintain photosystem stability (Peng et al., 2006). Photosystem I (PSI) reaction centre subunit IV (BVRB_7g166490) is capable of stabilizing the interaction between PsaC and the PSI core, assisting in the docking of iron oxidation reduction protein to PSI and interacting with iron oxidation reduction protein-NADP oxidoreductase (Dutta et al., 2014).

### 4.4 Potential impact on sugar beet breeding

Although conventional breeding can improve the performance of sugar beet in drought tolerance, artificial populations are limited by population size, insufficient recombination, and often large locus intervals, making selection both time-consuming and inefficient (He et al., 2010). Most of the natural populations used here have been reproduced over long periods of time and multiple generations, with adequate genomic recombination and accurate linkage localization, and these new genomic resources will facilitate genomic evolution in sugar beet and provide a scientific and theoretical basis for relevant research in drought tolerance.

## 5 Conclusion

In this study, 11 loci potentially associated with drought tolerance in sugar beet were screened using a mixed linear model (MLM). Genome-wide association analysis combined with qRT-PCR revealed that 13 genes were significantly differentially expressed between the two extreme groups of materials, with *BVRB_7g166480* being 46.69-fold more expressed in the drought-tolerant group than in the drought-sensitive group. It prompted us to further investigate the role of these interesting genes in sugar beet drought tolerance improvement in the future.

## Data Availability

The datasets presented in this study can be found in online repositories. The sequencing data has been uploaded. The link is https://www.ncbi.nlm.nih.gov/bioproject/PRJNA948801. The accession no. is PRJNA948801.
