# Correction: Genome-wide association study of drought tolerance traits in sugar beet germplasms at the seedling stage

**DOI:** 10.3389/fgene.2025.1685028

**Published:** 2025-10-24

**Authors:** Wangsheng Li, Ming Lin, Jiajia Li, Dali Liu, Wenbo Tan, Xilong Yin, Yan Zhai, Yuanhang Zhou, Wang Xing

**Affiliations:** ^1^ National Beet Medium-Term Gene Bank, Heilongjiang University, Harbin, China; ^2^ Key Laboratory of Sugar Beet Genetics and Breeding, College of Advanced Agriculture and Ecological Environment, Heilongjiang University, Harbin, China; ^3^ Xinjiang Academy of Agricultural Sciences, Urumqi, China

**Keywords:** sugar beet germplasms, PEG-6000 drought stress, SNP, GWAS, linkage disequilibrium

The **Data Availability statement** was erroneously given as “The datasets presented in this study can be found in online repositories. The sequencing data has been uploaded. The link is “https://www.ncbi.nlm.nih.gov/sra/PRJNA935487”. The accession no. is PRJNA935487.”

The correct Data Availability statement is “The datasets presented in this study can be found in online repositories. The sequencing data has been uploaded. The link is https://www.ncbi.nlm.nih.gov/bioproject/PRJNA948801. The accession no. is PRJNA948801.”

The original article has been updated.

